# Macrophage activation syndrome in a newborn: report of a case associated with neonatal lupus erythematosus and a summary of the literature

**DOI:** 10.1186/s12969-021-00500-w

**Published:** 2021-02-10

**Authors:** Veerle Heijstek, Meelad Habib, Roel van der Palen, Remco van Doorn, Petra Hissink Muller

**Affiliations:** 1 Department of Pediatric Rheumatology, Leiden University Medical Center, Leiden, the Netherlands; 2 Department of Dermatology, Leiden University Medical Center, Leiden, the Netherlands; 3 Division of Pediatric Cardiology, Department of Pediatrics, Leiden University Medical Center, Leiden, the Netherlands

**Keywords:** Neonatal lupus erythematosus, Complete AV block, Macrophage activation syndrome, Hemophagocytic lymphohistiocytosis

## Abstract

**Background:**

Macrophage activation syndrome (MAS) is a life-threatening hyperinflammatory syndrome and is caused by a severely dysregulated immune response. It has rarely been associated with neonatal lupus.

**Case presentation:**

We present a female neonate with MAS born to a mother who had cutaneous lupus erythematosus with circulating anti-nuclear antibodies (ANA), anti-SSA, anti-SSB and anti-extractable nuclear antigen (anti-ENA) antibodies.

Because of neonatal lupus (NLE) with a total atrioventricular block, epicardial pacemaker implantation was required on the sixth day of life. Following surgery she developed non-remitting fever and disseminated erythematous skin lesions. A diagnosis of MAS was made based on these symptoms, with hyperferritinemia, elevated transaminases, hypertriglyceridemia, and a skin biopsy that showed hemophagocytosis. Our patient was treated with steroids for 3 months with good effect. No relapse has occurred.

**Conclusions:**

MAS is a rare complication of neonatal lupus that may be difficult to diagnose, but needs to be treated promptly. In this article, pathogenesis and overlap of MAS and hemophagocytic lymphohistiocytosis (HLH) has been described.

Diagnosis of MAS can be difficult. Different diagnostic criteria are used in both diagnosing MAS and HLH. Validated criteria for diagnosis of MAS in other disease than systemic onset JIA have not been validated yet. In NLE, diagnosing MAS is even more difficult, since skin lesions are already common in NLE. We show the potential additional value of skin biopsy in diagnosing MAS.

## Background

Neonatal lupus erythematosus (NLE) is an auto-immune disease caused by transplacental transfer of maternal auto-antibodies anti-SSA (anti-Ro) and anti-SSB (anti-La) [1]. The risk of NLE in maternal auto-immune disease with these antibodies is approximately 2 % [1]. Severity of disease in the mother is not associated with severity of disease in the child. Mothers may have Systemic Lupus Erythematosus (SLE), subacute cutaneous lupus erythematosus, Sjögren syndrome or may not have any signs of auto-immune disease.

A well-known clinical presentation of NLE is neonatal cutaneous lupus and congenital complete heart block; NLE can also present with hepatitis, cytopenias and neurological abnormalities [2]. Macrophage activation syndrome (MAS) is a life-threatening hyperinflammatory syndrome and is caused by a severely dysregulated immune response. It has rarely been associated with neonatal lupus. So far, only four patients have been reported in the literature (Table 1) [6–9].

**Table 1:** Macrophage activation syndrome in neonatal lupus erythematosus - description of four cases in the literature

Sex	Antibodies	Clinical signs	Maximum Ferritin Ng/ml	Aminotransferases IU/L	Hematology	Triglycerides	Interleukins	Genetics	Therapy	Outcome	Reference
M	Anti Ro/SSA Anti La/SSB	22 h after birth: Complete AV-block Cardiopulmonary resuscitation	9769 (32 h)	ASAT 1027 ALAT 121 LDH 3490	Trombopenia day 24 (90 × 10^3)	-	Sol IL-23230 U/ml	HLH-genes negative	Hydrocortisone Relapse day 24th Hydrocortisone till 3 months	Severe psychomotor retardation	Suzuki (2013) [3]
M	Anti Ro/SSA Anti La/SSB	Directly after birth: Skin: annular plaques	660	ASAT 257 LDH 633	Trombopenia (104 × 10^3)		Sol IL-22280 U/ml		Prednisolone 1 mg/kg day 8 till 6 months	Good	Shimozawa (2015) [4]
F	ANA Anti Ro/SSA	10 days after birth: Tachypnoea, Fever Hepatosplenomegaly	Maximum 2891	Maximum day 10 ASAT 459 ALAT 463	Anemia (12.8 mg/dl) Trombopenia lowest 12 (day 18)	Day 10: 280 mg/dL			IVIGS (1 g/kg 2 days) Methylprednisolone pulse (HLH protocol 2004) Relapse day 26: Ciclosporin 6 mg/kg. 54 day’s steroids	2 months: good	Park (2015) [5]
F	ANA Anti ENA Anti Ro/ SSA Anti SSB/La	Fever Skin lesions (annular plaques)	Maximum 4439	Maximum ASAT 84 ALAT 33 LDH 909	Day 5 Trombocytes 135 Hb lowest 7.1 mmol/L day 32	3.88 mmol/L	Sol IL-221803 pg/ml	HLH-genes negative	Prednisolone 1 mg/kg` Tapered down in 6 months	Good Antibodies negative after 6 months	2020 (our case)

Although MAS is a well-known complication of systemic-onset juvenile idiopathic arthritis (soJIA) it less frequently occurs associated with other autoimmune diseases such as Kawasaki disease, systemic lupus erythematosus, polyarticular juvenile idiopathic arthritis, juvenile dermatomyositis, anti-phospholipid syndrome and mixed connective tissue disease [10–13]. MAS is considered to represent a secondary form of hemophagocytic lymphohistiocytosis (HLH). Diagnosing MAS in diseases other then soJIA is challenging, because diagnostic criteria are only validated in soJIA. Skin findings are not included in the diagnostic criteria for (primary) hemophagocytic lymphohistiocytosis (HLH) or in MAS [14]. However, skin eruption can be an important clinical finding in patients with HLH.

Primary HLH is a group of autosomal recessive immune disorders, all leading to a life-threatening hyperinflammatory state. It is linked to various genetic defects, mostly affecting the perforin-mediated cytolytic pathway [10, 11]. Perforin is a protein, necessary for inducing apoptosis of target cells (viruses, tumors) [15].

It has been suggested that MAS, primary and secondary forms of HLH are a spectrum of the same disease rather than single entities [10, 16], and steps have been made to think of MAS as ‘reactive’ HLH. Genetic studies confirm overlap between these entities.

In this case report, we show a case of MAS as a complication of NLE, potentially triggered by infection and maybe surgery as well [17], without a known HLH-susceptibility gene mutation. Additionally, we present a review of the literature of the pathophysiology of MAS and HLH in NLE patients.

## Case

A 29-year old, secondary gravida female was referred to the obstetrics department. She was diagnosed with cutaneous lupus erythematosus several years before pregnancy. She had positive anti-nuclear antibodies (ANA), anti-extractable nuclear antigen (anti-ENA), anti-SSA and anti-SSB. Treatment before pregnancy consisted of topical steroids. Before pregnancy, she was never treated with systemic therapy. During gestation she was asymptomatic without medication. Pregnancy was followed carefully at our obstetrics department because of her condition. A structural ultrasound examination at 20 2/7 weeks of gestation showed a fetal bradycardia (50 beats per minute (bpm)), based on a complete atrioventricular (AV) block in absence of a structural heart disease. No other structural abnormalities were found and no hydrops was present. Although controversial and a matter of debate [2, 18], mother was treated after diagnosis of the AV block with dexamethasone in order to suppress a potential ongoing inflammation to prevent fibrotic replacement in the AV node. No improvement of the fetal heart block was observed during close follow-up.

**Fig. 1:**
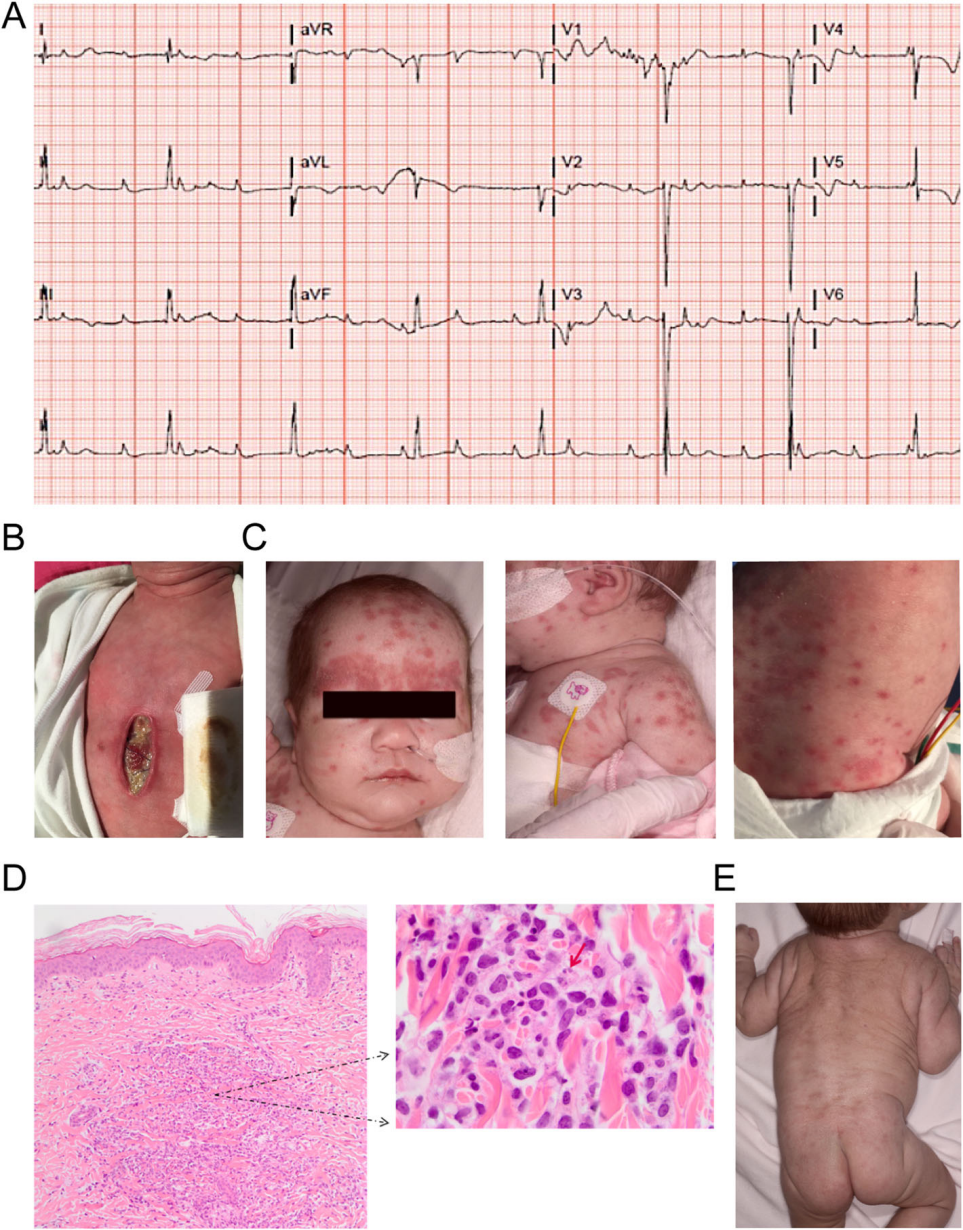
**a** ECG: third degree AV block with junctional escape rhythm (~50 bpm), atrial rate ~115 bpm. **b** Day 12: Midsternal pacemaker pocket wound 7 days after surgical exploration for infection, pacemaker in situ. **c** Day 22: Numerous round to oval and partly confluent erythematous papules and plaques, located on the forehead and eyebrows, as well as on the trunk and extremities were noted. **d** Pathology of skin biopsy: A dense dermal (perivascular) infiltrate is shown on the left. The red arrow points to a hemophagocytic macrophage. **e** Resolving skin lesions, four weeks after start of treatment

At 37 2/7 weeks of gestation, a female infant was born, birth weight was 2330 g (p5) and APGAR scores 9/10. Complete AV block was confirmed and there was a junctional escape rhythm of 50 bpm (Fig. 1a). The child was admitted to our neonatal intensive care unit for clinical observation. Due to circulatory insufficiency caused by the total AV block with a junction escape rhythm of 50 bpm, isoprenalin was started. Implantation of an epicardial VVI pacemaker system in abdominal position (Microny™^,^ St Jude Medical) was inevitable on day 6.

Two days later, the patient developed fever and the pacemaker implantation wound appeared erythematous and inflamed. Blood- and wound cultures acquired before and during antibiotic treatment remained sterile. C-reactive protein (CRP) was maximum 80 mg/L on day 9 post-birth (ref < 5 mg/L). She was treated for a suspected wound infection with flucloxacillin and gentamycin intravenously. Furthermore, she was treated for perianal candidiasis with topical application of miconazole cream. Despite antibiotic therapy, there was an ongoing redness around the pacemaker pocket with cloudy, purulent fluid draining from the wound and fever persisted, one week after pacemaker implantation.

Surgical wound exploration was performed showing infiltrates with pus, for which antibiotic treatment was switched to ceftazidime. The pacemaker system was left inside and the wound was left open. In the days after exploration the redness slightly diminished around the elevated wound edges with granulation tissue appearing in the wound bed (Fig. 1b). CRP values remained low; varying between 6 and 13 mg/L. In between, fever persisted. The patient developed a generalized non-pruritic skin eruption on the 22nd day of life. Numerous round to oval and partly confluent erythematous papules and plaques, located on the forehead and eyebrows, as well as on the trunk and extremities were noted (Fig. 1c). Some papular lesions had an erosive center. The oral mucosa, palms and soles were not affected. There was no hepatosplenomegaly or lymphadenopathy.

Persistent fever, despite antibiotic treatment, and the atypical extensive skin lesions prompted us to think of other diagnosis like MAS. Our differential diagnosis consisted of persistent infection (systemic candidiasis, CMV, EBV varicella, (perianal skin culture positive for *Candida albicans*. CMV and EBV both (maternal) IgG positive, IgM negative)); auto-immune (complex neonatal lupus) or auto-inflammatory disease (MAS, primary HLH) or a primary immunodeficiency.

Additional blood tests showed a ferritin level of 4162 mug/L (ref. 10–150 mug/L), a mild anemia (Hb 7.1 mmol/L; 114.4 g/L; ref. 8.5–12.5 mmol/L), leukocytes and thrombocyte counts remained normal. Fibrinogen was 3.6 g/L (ref. 2.1–3.8 g/L), D-dimer 3996 ng/ml; ref. < 500 ng/ml). Furthermore, mildly elevated aminotransferase levels (ASAT 84 U/L (ref. < 31 U/L), ALAT 33 U/L (ref. < 34 U/L), LDH 909 U/L (ref. < 600 U/L) and elevated triglyceride levels, (343.7 mg/dl; ref. < 77.3 mg/dl) were reported. Similar to her mother, she had positive antibodies: ANA, anti-ENA, anti-SSA and B. Soluble IL-2 receptor level was 21,803 pg/ml (< 2500 pg/ml). These results are shown in Table 2.

**Table 2:** Laboratory values of our patient

Laboratory parameter (reference / unit)	Day 9	Day 29	Day 31 (+ 1 after start prednisolone)	Day 33 (+ 3)	Day 37 (+ 7)	Day 44 (+ 14)	Day 65 (+ 35)	Day 97 (+ 67)	Day 191	Day 260
CRP (< 5 mg/L)	78.8	12	7.5	< 3	< 3	< 3	< 3	< 3		
Hemoglobin (8.5–12.5 mmol/L)	12.3	8.4	8.0	7.1	7.5	7.5	7.9	8.4	7.7	8.0
Ferritin (10–150 mug/L)		4162	4439	2465	1834	828	169	138	29	29
Leukocytes (6-17 × 10^9/L)	12.53	10.7	6.14	7.86	8.99	8.64	7.85	8.12	14.94	11.3
Thrombocytes (150-600 × 10^9/L)	166	195	187	269	372	543	557	332	532	468
Fibrinogen (2.1–3.8 g/L)			3.6	3.0	3.2					
D-dimer (< 500 ng/ml)			3996	2977	1921					
ASAT (< 31 U/L)		79	84	57		28	38	53		
ALAT (< 34 U/L)		31	33	31		28	66	57		
LDH (< 300 U/L)		810	909	742		368	266	369		
Triglyceride (0.8–2.3 mmol/L)		1.73	2.49	3.88	3.72					
ANA (−)		+						+	Weakly positive	–
Anti ENA (< 0.7 U/ml)		21.0						9.2	0.5	0.1
Anti SSA (< 7.0 U/ml)		24.0						10	–	–
Anti SSB (< 7.0 U/ml)		> 320						187	–	–
Soluble-IL2R (< 2500 pg/ml)			21,803							

Because of the non-remitting high-grade fever, extensive skin rash, in combination with liver enzyme abnormalities, elevated ferritin and proven NLE, MAS complicating NLE was suspected and a skin biopsy was taken to exclude other diseases as mentioned previously. Histological examination of a skin biopsy from a lesion on the trunk revealed a dermal interstitial and perivascular infiltrate, mainly consisting of histiocytes. The most notable finding was the presence of hematophagocytosis in the dermis (Fig. 1d). No pathological alterations were found in the epidermis and there was no histopathological evidence of an interface dermatitis and scantly periadnexal infiltration, as can be observed in cutaneous lupus erythematosus. In the skin biopsy sample we studied there was no evidence of involvement of the subcutis, as can be observed in cytophagic histiocytic panniculitis.. Hemophagocytosis describes the histopathological finding of phagocytosis of erythrocytes, platelets, leukocytes and their precursors by histiocytes. Hemophagocytosis in this skin biopsy strongly supports the diagnosis of MAS complicating NLE.

With a presumptive diagnosis of MAS complicating NLE, potentially triggered by wound infection and a pro-inflammatory state after surgery, treatment with prednisolone (2 mg/kg) was started, with vitamin D suppletion. Within 24 h after prednisolone was started, fever subsided. In 2 weeks, ferritin levels dropped and normalized within 2 months. Skin lesions resolved (Fig. 1e) and the pacemaker pocket wound healed completely in 4 weeks. Prednisolone was tapered slowly in 6 weeks and stopped after 3 months of treatment. At that moment, ferritin level was normalized, ANA and anti-ENA remained positive, but anti SSA/B titers were undetectable.

Follow up after 6 months demonstrated a positive ANA, but negative anti ENA, negative anti SSA and negative anti SSB. ANA became negative after 8 months. Genetic tests (next generation sequencing, gene panel) were performed to differentiate between primary HLH or MAS and secondary HLH. No mutations in nine HLH susceptibility genes (*PRF1, UNC13D, STX11, STXBP, SH2D1A, XIAP, LYST, RAB27A, AP3B*) [19] were detected.

## Discussion

It has been suggested that MAS, primary and secondary forms of HLH are a spectrum of the same disease rather than single entities [10, 16], and steps have been made to think of MAS as ‘reactive’ HLH [10]. In the spectrum of MAS/HLH, due to genetic susceptibility, a trigger (infection, auto-immune disease, malignancy) results in excessive activation and expansion of monocytes and macrophages; with high levels of circulating cytokines (IFN-gamma/type II interferon-response); IL-2; IL-1, IL-6, IL-18 and TNF-alpha; and also cytokine inhibitors such as soluble TNF-receptors [10]. IL-18 seems to be particular relevant in MAS, since strikingly high levels have been observed in patients with MAS in soJIA. IL-18 is believed to drive the action of CD8+ T-cells and their production of IFN-g, enhancing the cytokine storm [4, 10]. In MAS, IL-1 and IL-18 are upregulated [4].

In a pro-inflammatory state, cytolytic cells are important in termination of immune responses by inducing apoptosis. The following mechanism of the cytokine storm in MAS and HLH is suspected: the failure of the apoptosis induction of target cells by cytolytic cells might delay the contraction stage of the immune response, leading to persistent expansion of activated T-lymphocytes, macrophages and excessive and persistent cytokine release [10]. Failure to induce apoptosis, due to cytotoxic dysfunction leads to the cytokine storm. In such cytokine storm, clinical findings are non-remitting fever, cytopenias, hepatosplenomegaly and coagulation disorders with extremely high levels of ferritin and hypertriglyceridemia.

In primary HLH, several genetic defects have been described. Most predisposing genetic defects (15–40%) are in the perforin-mediated cytolytic pathway [15, 20]. In about 10–30% of primary HLH, the disease is caused by mutations in MUNC13–4 gene [15, 20]. With this mutation, the function of cytolytic cells is diminished, although the production of perforin is normal. Other genetic mutations related to primary HLH are STXBP2 (syntaxin binding protein 2) and syntaxin 11 [21]. These genes are necessary in the pathway of the release of perforin.

In secondary HLH or MAS, different mutations have been found in previously mentioned genes (Perforin 1, MUNC13–14, STXBP2, Syntaxin 11). Heterozygous mutations have been found in up to 76% of individuals with secondary HLH and MAS [21, 22] as well as mutations in non-coding regions and splice-site mutations of HLH genes, which correlates with a less severe disease course and a later age of onset [21].

A threshold model has been proposed in MAS, in which combinations of genetic predisposition, underlying inflammatory state and triggering infectious agents, results in a clinically relevant cytokine storm syndrome and MAS [10, 21, 23].

### Skin lesions

Skin lesions are not included in the diagnostic criteria for (primary) hemophagocytic lymphohistiocytosis (HLH) or in MAS [14]. However, skin eruption can be an important clinical feature in patients with HLH. According to the literature, 6–65% of patients with HLH have skin involvement during the course of disease [3, 5, 24]. Also in MAS/HLH, skin findings have been reported [7, 25, 26]. Cutaneous manifestations of MAS/HLH are however aspecific as most patients present with a transient generalized maculopapular eruption. Patients may also develop skin eruptions that are related to the additional effects of the MAS/HLH (e.g. thrombocytopenic purpura) [3, 27].

In general, skin lesions in neonatal lupus appear after exposition to natural UV-light, in the first 2 months after birth. Skin abnormalities earlier in life, even directly after birth, have been described as well [28]. The pathology of the skin biopsy in our case did not show mononuclear cell infiltration and immunoglobulin deposition, as is expected in neonatal lupus skin abnormalities. Although performing bone marrow aspirate and biopsy is part of the diagnostic work-up of MAS/HLH, less is known about the clinical relevance of skin biopsy in the work-up for MAS/HLH. The classical histopathological skin findings of MAS/HLH include a lymphohistiocytic (perivascular) infiltrate in the dermis without epidermal abnormalities. The most important finding is the presence of hemophagocytosis. This term describes the histopathological finding of phagocytosis of erythrocytes, platelets, leukocytes and their precursors by histiocytes [27]. Data on the presence of hemophagocytosis in a skin biopsy in MAS are lacking, but it has been described before in case reports, with one case with cutaneous hemophagocytosis in NLE [29, 30]. The presence of hemophagocytosis is one of the key features of MAS, as can be seen in bone marrow biopsy in up to 60% of patients with MAS secondary to soJIA [31]. In our case, skin biopsy instead of bone marrow aspirate confirmed the diagnosis of MAS in NLE.

### Diagnosis of MAS/HLH

In general, it can be challenging to diagnose MAS/HLH in either primary or secondary forms. Clinical and laboratory features of MAS include sustained fever, hyperferritinemia, pancytopenia, fibrinolytic consumptive coagulopathy and liver dysfunction. There is no diagnostic test or a set of disease uniform diagnostic criteria to differentiate MAS from the underlying systemic inflammatory condition. An expert consensus panel published validated diagnostic criteria for MAS complicating soJIA in 2016 [32]: persistent fever in a patient with soJIA with serum ferritin level > 684 ng/ml plus any of the following: platelet count < 181*10^9/l, ASAT > 48 units/l, triglyceride concentration > 156 mg/dl or fibrinogen < 360 mg/dl [12].

For secondary HLH, with similar clinical spectrum as MAS, more validated diagnostic criteria are noted in the HLH-2004 diagnostic guidelines [14]. At least five of the following eight criteria are required for the diagnosis: fever, splenomegaly, cytopenias (> 2 of 3 cellines), hypertriglyceridemia (> 265 mg/dl) and/or hypofibrinogenemia (< 1.5 g/l), hemophagocytosis in bone marrow or spleen or lymph nodes, low or absent natural killer cell activity, ferritin > 500 μg/L and sCD25 > 2400 units/ml. The HLH-2004 criteria may delay diagnosis in patients with a less severe initial presentation, since three of eight criteria for diagnosis (sCD25, NK cell function, evidence of hemophagocytosis on bone marrow biopsy) are not fast and easily available.

Our patient fulfilled the diagnostic criteria of MAS secondary to soJIA [32] but did not meet the criteria for the HLH-2004 criteria: only 4 out of 8 criteria were present (i.e. fever, hyperferritinemia, hypertriglyceridemia. Skin biopsy confirming hemophagocytosis instead of bone marrow biopsy; of note: NK-cell activity was not evaluated).

Levels of IL-18, reflecting inflammasome activation [33], have been reported to be markedly increased in MAS and only moderately elevated in other rheumatic disease and in familial HLH [4, 33]. It possibly reflects the extent of macrophage activation, given that macrophages seem to be the main source of IL-18. Levels of IL-18 correlate with levels of ferritin [33]. Currently, it can be measured but it is not widely available yet and was not measured in our patient [4, 10, 12]. High levels of IL-18 have been found in neonates born to a mother with adult onset Stills disease [9].

Next to their antiviral effects, growing evidence suggests that type I and II interferons have immunoregulatory functions that are critical for dampening immunopathogenic mechanisms and minimizing collateral damage from the infection [34].

For example, the type I interferon family has a central role in the pathogenesis of several autoimmune diseases as in SLE and RA [35]. MAS and HLH are known as type II interferonopathies [34] (ref dual nature). The type I and II interferon response are closely related and the type I interferon response is capable of suppressing the type II response [34].

In our patient; we hypothesize primary activation of the type I response (because of maternal auto-antibodies anti SSA, anti SSB). Wound infection and possibly (yet unproven) genetic susceptibility leaded to a type II interferon response.

It would have been informative to measure these interferon responses but has not been done in our patient since it was not available yet at that time.

### Treatment

MAS/HLH secondary to NLE has been treated with steroids as monotherapy in the few cases that has been reported (Table 1). In two neonates, a relapse occurred when steroids were tapered and stopped after 4 weeks of treatment. Longer treatment, around three to 6 months seems to be necessary. This might be related to the disappearance of maternal antibodies in children within the first 3 months after birth.

There are no validated treatment guidelines on MAS in soJIA, nor in other rheumatological disease. First-line treatment is often started with high-dose corticosteroid therapy. Cyclosporine A (an immunosuppressive drug which acts in lowering T-lymphocytes) is also reported to induce remission, as monotherapy or as part of a combination regimen. Etoposide has been given as well, as it is part of the HLH-2004 protocol. IL-receptor antagonists, such as Anakinra, are increasingly used in the treatment of MAS in soJIA, especially in steroid-resistant MAS [10, 12, 36, 37]. Since IL-18 seems to play an important role in the development of MAS, IL-18 blockage may be an important therapeutic target in future therapy, as is currently under study in NLRC4-MAS inflammasomopathies (ClinicalTrials.gov Identifier: 113760). Recently, promising results in the treatment of primary HLH have been shown in a phase 2–3 trial, in which emapalumab (a human anti-interferon-y antibody); administered with dexamethasone, showed to be effective in the treatment of primary HLH [38]. This monoclonal antibody binds interferon-y and neutralizes its biologic activity,controlling hyperinflammation.

MAS/HLH in rheumatic disease (soJIA, Kawasaki, SLE) has a more favourable outcome than in primary HLH, which relies on more treatable factors. When the auto-immune/inflammatory disease is treated properly, the trigger for cytokine storm will be gone [19]. Primary HLH needs to be treated following intensive HLH-2004 guidelines [14], this is outside the scope of this article to address in further detail.

Our patient has been followed for the first 3 years. She had a growth restriction; her development is normal. The complete AV block persisted and the implanted pacemaker device is working properly. The trigger for MAS/HLH is gone, autoantibodies have become negative. Although long-term effects of NLE complicated by HLH/MAS are not known, no important sequellae have been observed so far.

## Conclusion

In children with NLE or in children born from mothers who are SSA- and/or SSB-positive, every condition with persistent fever, abnormal wound healing, atypical skin eruption, should raise awareness of the possible diagnosis of MAS/HLH complicating NLE.

Recent diagnostic criteria of MAS in soJIA seem to be useful in more rheumatological diseases, for instance NLE, but have not been validated yet. A lesional skin biopsy can be taken to assess the presence of hemophagocytosis in the dermal compartment. The results of a skin biopsy may add in the diagnostic work-up and may lead to treatment at an earlier stage.

## Data Availability

Not applicable.

## Abbreviations

ANA: Anti-nuclear antibodies
Anti-ENA: Anti-extractable nuclear antigen
Anti-SSA: Anti-Sjögren-syndrome-related antigen A / also called anti-Ro
Anti-SSB: Anti-Sjögren-syndrome-related antigen B / also called anti-La
AV block: Atrioventricular block
AV node: Atrioventricular node
CMV: Cytomegalovirus
CRP: C-reactive protein
EBV: Epstein-Barr virus
HLH: Hemophagocytic lymphohistiocytosis
JIA: Juvenile idiopathic arthritis
MAS: Macrophage activation syndrome
NLE: Neonatal lupus erythematosus
soJIA: Systemic-onset juvenile idiopathic arthritis
SLE: Systemic lupus erythematosus
LDH: Lactate dehydrogenase

## References

[1] Klein-Gitelman MS (2016). Neonatal lupus: what we have learned and current approaches to care. Curr Rheumatol Rep.

[2] Vanoni F, Lava SAG, Fossali EF, Cavalli R, Simonetti GD, Bianchetti MG (2017). Neonatal systemic lupus erythematosus syndrome: a comprehensive review. Clin Rev Allergy Immunol.

[3] Morrell DS, Pepping MA, Scott JP, Esterly NB, Drolet BA (2002). Cutaneous manifestations of hemophagocytic lymphohistiocytosis. Arch Dermatol.

[4] Griffin G, Shenoi S, Hughes GC. Hemophagocytic lymphohistiocytosis: an update on pathogenesis, diagnosis, and therapy. Best Pract Res Clin Rheumatol. 2020;34(4):101515. 10.1016/j.berh.2020.101515. Epub 2020 May 7. 10.1016/j.berh.2020.101515 32387063

[5] Jun HJ, Kim HO, Lee JY, Park YM (2015). Preceding annular skin lesions in a patient with Hemophagocytic Lymphohistiocytosis. Ann Dermatol.

[6] Park JH, Kim SH, Kim HJ, Lee SJ, Jeong DC, Kim SY (2015). Macrophage activation syndrome in a newborn infant born to a mother with autoimmune disease. J Perinatol.

[7] Shimozawa H, Kono Y, Matano M, Suzuki Y, Koike Y, Yada Y (2015). Cytokine profile in two siblings with neonatal lupus erythematosus. Pediatr Int.

[8] Suzuki Y, Takahashi N, Yada Y, Koike Y, Matano M, Nishimura H (2013). Hemophagocytic lymphohistiocytosis in a newborn infant born to a mother with Sjogren syndrome antibodies. J Perinatol.

[9] Shimizu M, Kizawa T, Kato R, Suzuki T, Yachie A (2019). Macrophage activation syndrome in neonates born to mothers with adult-onset Still's disease: perinatal effect of maternal IL-18. Clin Immunol.

[10] Grom AA, Horne A, De Benedetti F (2016). Macrophage activation syndrome in the era of biologic therapy. Nat Rev Rheumatol.

[11] Sen ES, Steward CG, Ramanan AV (2017). Diagnosing haemophagocytic syndrome. Arch Dis Child.

[12] Crayne CB, Albeituni S, Nichols KE, Cron RQ (2019). The immunology of macrophage activation syndrome. Front Immunol.

[13] Lerkvaleekul B, Vilaiyuk S (2018). Macrophage activation syndrome: early diagnosis is key. Open Access Rheumatol.

[14] Henter JI, Horne A, Arico M, Egeler RM, Filipovich AH, Imashuku S (2007). HLH-2004: diagnostic and therapeutic guidelines for hemophagocytic lymphohistiocytosis. Pediatr Blood Cancer.

[15] Schulert GS, Grom AA (2015). Pathogenesis of macrophage activation syndrome and potential for cytokine- directed therapies. Annu Rev Med.

[16] Bracaglia C, Prencipe G, De Benedetti F (2017). Macrophage activation syndrome: different mechanisms leading to a one clinical syndrome. Pediatr Rheumatol Online J.

[17] Dabrowska AM, Slotwinski R (2014). The immune response to surgery and infection. Cent Eur J Immunol.

[18] DeNoble AE, Kuller JA, Rhee EJ (2015). Controversies in the Management of Isolated Congenital Atrioventricular Block. Obstet Gynecol Surv.

[19] Chinn IK, Eckstein OS, Peckham-Gregory EC, Goldberg BR, Forbes LR, Nicholas SK (2018). Genetic and mechanistic diversity in pediatric hemophagocytic lymphohistiocytosis. Blood.

[20] Ravelli A, Grom AA, Behrens EM, Cron RQ (2012). Macrophage activation syndrome as part of systemic juvenile idiopathic arthritis: diagnosis, genetics, pathophysiology and treatment. Genes Immun.

[21] Zhang M, Behrens EM, Atkinson TP, Shakoory B, Grom AA, Cron RQ (2014). Genetic defects in cytolysis in macrophage activation syndrome. Curr Rheumatol Rep.

[22] Kaufman KM, Linghu B, Szustakowski JD, Husami A, Yang F, Zhang K (2014). Whole-exome sequencing reveals overlap between macrophage activation syndrome in systemic juvenile idiopathic arthritis and familial hemophagocytic lymphohistiocytosis. Arthritis Rheumatol.

[23] Strippoli R, Caiello I, De Benedetti F (2013). Reaching the threshold: a multilayer pathogenesis of macrophage activation syndrome. J Rheumatol.

[24] Bay A, Calka O, Akdeniz N, Oner AF, Kirimi E (2006). Newborn infant with hemophagocytic lymphohistiocytosis and generalized skin eruptions. J Dermatol.

[25] Shwin KW, Lee CR, Goldbach-Mansky R (2017). Dermatologic manifestations of monogenic autoinflammatory diseases. Dermatol Clin.

[26] Li X, Qu B, Nie Y, Zhu G, Li W, Mu F (2014). Clinical features of macrophage activation syndrome in the adult northern Chinese population. Lupus..

[27] Santos-Arroyo A, Barrera-Llaurador J, Sanchez JE, Martin-Garcia R, Sanchez JL (2017). Role of skin biopsies in the diagnosis of Hemophagocytic Lymphohistiocytosis. Am J Dermatopathol.

[28] Cimaz R, Biggioggero M, Catelli L, Muratori S, Cambiaghi S (2002). Ultraviolet light exposure is not a requirement for the development of cutaneous neonatal lupus. Lupus..

[29] Chamseddin B, Marks E, Dominguez A, Wysocki C, Vandergriff T. Refractory macrophage activation syndrome in the setting of adult-onset still disease with hemophagocytic lymphohistiocytosis detected on skin biopsy treated with canakinumab and tacrolimus. J Cutan Pathol. 2019;46(7):528–31. 10.1111/cup.13466. Epub 2019 Apr 23. 10.1111/cup.13466 30927277

[30] Kerl K, Wolf IH, Cerroni L, Wolf P, French LE, Kerl H (2015). Hemophagocytosis in cutaneous autoimmune disease. Am J Dermatopathol.

[31] Cron RQ, Davi S, Minoia F, Ravelli A (2015). Clinical features and correct diagnosis of macrophage activation syndrome. Expert Rev Clin Immunol.

[32] Ravelli A, Minoia F, Davi S, Horne A, Bovis F, Pistorio A (2016). 2016 classification criteria for macrophage activation syndrome complicating systemic juvenile idiopathic arthritis: a European league against rheumatism/American College of Rheumatology/Paediatric rheumatology international trials organisation collaborative initiative. Arthritis Rheumatol..

[33] Canna SW, Marsh RA (2020). Pediatric hemophagocytic lymphohistiocytosis. Blood..

[34] Lee AJ, Ashkar AA (2018). The dual nature of type I and type II interferons. Front Immunol.

[35] El-Sherbiny YM, Psarras A, Md Yusof MY, Hensor EMA, Tooze R, Doody G (2018). A novel two-score system for interferon status segregates autoimmune diseases and correlates with clinical features. Sci Rep.

[36] Boom V, Anton J, Lahdenne P, Quartier P, Ravelli A, Wulffraat NM (2015). Evidence-based diagnosis and treatment of macrophage activation syndrome in systemic juvenile idiopathic arthritis. Pediatr Rheumatol Online J.

[37] Mehta P, Cron RQ, Hartwell J, Manson JJ, Tattersall RS. Silencing the cytokine storm: the use of intravenous anakinra in haemophagocytic lymphohistiocytosis or macrophage activation syndrome. Lancet Rheumatol. 2020. 10.1016/S2665-9913(20)30096-5 PMC7198216 32373790

[38] Locatelli F, Jordan MB, Allen C, Cesaro S, Rizzari C, Rao A (2020). Emapalumab in children with primary Hemophagocytic Lymphohistiocytosis. N Engl J Med.

